# Disclosures for slavery accounting concerning SDG 8 and corporate attributes: a study on the banking industry of Bangladesh

**DOI:** 10.1186/s43093-023-00199-z

**Published:** 2023-05-03

**Authors:** Trina Saha, Rubel Miah, Mahfujur Rahman, Sumon Kumar Das, Emranul Hoque

**Affiliations:** 1 Department of Business Administration, Noakhali Science and Technology University, Sonapur, Noakhali 3814 Bangladesh; 2 Comilla University, Salmanpur, Bangladesh; 3 Bangabandhu Sheikh Mujibur Rahman Maritime University, Mirpur, Bangladesh

**Keywords:** Slavery accounting, SDGs, Corporate governance, Profitability, Banks, Bangladesh

## Abstract

With the continuous adoption of sustainable development goals by all countries, there is a rising demand for implementing and disclosing related information by companies. This paper aims to find an idea about the nature of reporting practices related to slavery by listed banking companies of Bangladesh and the relationship between reporting practice and organizational attributes. Existing literature provides the foundation of this study. Annual reports from 2016 to 2021 were collected from the company websites, and content analysis was used to determine the nature and extent of slavery reporting; an index was developed based on content analysis. Independent variables were determined based on the current literature review. Statistical tools, including the test of multicollinearity, heteroscedastic, correlation, and linear regression and panel data analyses were used to determine the fitness of the model and the impact of independent variables on the dependent variable. Content analysis showed a clear picture of the consciousness about slavery accounting as all of the companies reported a minimum of three components of selected targets from SDG8. The evidence says that the quality and quantity of slavery reporting are improving yearly. A satisfactory correlation was found among the variables. Some variables, such as ownership nature, Board Size, etc., have a positive impact, and insignificant impact was found for Age and leverage on slavery disclosure. This paper only examines the banking industry, and it assesses only a few targets of SDG 8. Thus the results obtained from the study may not be similar to other companies. Data collection also has limitations; our target was to study till 2022 but some information requirements of few companies were not available on websites. This research paper is the first attempt to determine the nature of slavery accounting in Bangladesh. It will encourage business organizations to extend their reporting on slavery and SDGs.

## Introduction

Whenever we hear the word 'slavery,' a picture of one human who is deprived and treated badly like a bonded property by another comes to mind. However, slavery has changed its outlook but left its trace across cultures and countries. Despite several human rights laws, it exists more or less in every culture behind forced labor, child labor, bonded labor, forced marriage, human trafficking, etc. Caruana et al. [12] defined modern slavery as situations when persons cannot leave or refuse in fear of threats, economic coercion, violence or abuse of power. They identified it as a societal problem that grabbed the attention of policymakers, the general public, business leaders, and governments. Voss et al. [51] expressed modern slavery as an umbrella term circulates in business policy, civil society, and legislative areas in the face of various forms of exploitation, such as labor exploitation, human trafficking, forced marriage, etc.

People, especially females and children, are being trapped in the hedge of modern slavery because of poverty and globalization. It exists whenever a person is compelled to do something by force, coercion, or fraud. Modern slavery is everywhere, from the colorful, secured cities of developed countries to the less secured countries immersed in hunger. It occurs more in industries, basically labor-intensive and under-regulated sectors that are trying to move production section to countries where cheap labor is available, especially in the least developed countries. Gold et al. [19] explored the management tools and indicator systems for detecting slavery, such as percentage of poor performance, insufficient employment opportunities, exploitation of workers, and a high percentage of migrants, minorities, and responses to manage it after detection. They suggested three approaches to mitigate the use of slave labor: multi-stakeholder initiative, community-centered strategy, and supplier development and capacity building. Several research mainly focused on labor deregulation, non-payment of compensation, or minimum wage that must be properly controlled by employment authorities and agencies.

Due to COVID 19 pandemic, most industries went on lockdown, increasing vulnerable people searching for work that works as a key driving force behind slavery-related fraudulent activities. It has now become a global challenge that is impossible for any government of its own to beat. Businesses are currently engaged in the complex supply chain resulting greater level of human exploitations more than ever. This can result in loss of reputation, trust of consumers, legal consequences, and financial loss. It is now a demand from customers, regulatory agencies, and governments to address slavery in supply chains and business operations. Policies are followed by a top-down approach, which ignores the key players; thus, strong moral pressure for anti-slavery disclosure is needed for promoting policies. There are still questions about social, political, and legal contexts encouraging favorable situations for increasing slavery [10]. Mere regulations are not a solution to reducing modern slavery; awareness and corporate behavior changes are also needed. Regulations can play a major role in portraying the disclosure systems of accounting firms [37, 43].

Sustainable Development Goals are a complete outline aiming to gear up our most demanding social, economic, and environmental challenges on the road to 2030. The Millennium Development Goals that came before mainly focused on developing countries; however, SDGs work as a community approach where every country works as a family. These are the big goals to reduce gender inequality in poverty, hunger, education, climate change, and social justice by 2030 that can be achieved by working together. Initially, it was not a priority to eradicate slavery, but now this plan also addresses exploitation, trafficking, and violence against humans. As it applies equally to every single country, no matter what their location or what their context, Government, business organizations, people, in a word, all sectors of all country need to stay and work together under one roof.

Moussa et al. [35] made a relationship between modern slavery and sustainable development goals. They found a negative relationship between institutional environment quality and modern slavery risk. The business organization is leading in achieving SDGs by creating jobs, ensuring a healthy environment for workers, and addressing sustainability in using scarce resources [34]. Now, sustainability has become a global agenda; it combines environment, ethics, and economics, creating a new attitude toward each other and toward nature. As it is multidisciplinary, there is great room for research in political economy and social sciences. And accountants play a major role in integrating these goals into each phase of operations and decision-making. Makarenko and Plastun [31] said that incorporating SDGs at all levels of the decision-making process and business operations and communicating these with interested stakeholders through sustainable reporting are critical tasks for accountants. There are the value creators as they are implementing SDGs in strategies, policies, and activities of management; they are the value providers as they provide high-quality reports based on a sustainable approach to the administration; they are the value keepers as they protect all kinds of capital including financial, social, natural and industrial capital of the company. And finally, they are the value reporters as they are the only responsible party for ensuring high-quality reporting for all kinds of stakeholders. The UK slavery act works like a bridge with government priorities and highlights the impact of government initiatives on vulnerable migrant workers [43]. Recent legislation developments now focus on industries that show carefulness in reporting human rights violations playing a leading role. Nolan [36] conducted this study on corporate responsibility to human rights, particularly focusing on global supply chains as this has been connected with a wide range of human rights violation cases. The problem arises in regulating this kind of chain because there is no beginning and end of boundaries.

In Bangladesh, as it has already achieved MDGs, business organizations are now being aware of SDGs; they are addressing sustainable factors in their annual report, sustainability report, integrated report, etc. The picture was different few years back. Basically after 1990s, several stakeholder group has become concerned about social performance of companies operating in this country. They work as a driving force behind social-related reporting and disclosures [7] Disclosing information related to the safeguard of human rights encourages investors to invest more in responsible business organizations. Transparency in corporate behavior that can improve and protect human rights, draw information about human rights policies and encourage open analysis from stakeholders [28]. Organizations should focus on the betterment of the knowledge of supply chain to combat slavery in the working environment [44]. Several studies have been carried out on financial reporting, social reporting, sustainability reporting. To the authors’ best knowledge this is the first study to correlate SDGs with slavery accounting in Bangladesh perspectives. This study aims to find out how the listed companies are addressing slavery accounting in their report within the framework of SDG by asking the following research questions:

- RQ 1: Do the listed companies account for slavery in their annual report?
- RQ 2: What are the factors impacting the disclosure for slavery?

To find out the answers, we have selected Banking companies as samples as this industry is working hard in terms of the safety of the working environment. This industry also play a major role in creating and maintaining a favorable investment environment in the country [11]. The rest of the paper includes Sect. "Literature review and hypothesis development; Sect. "Research design and methodology"; Sect. Results and discussions; Sect. Conclusions; & Sect.  Limitations and future research direction.

## Literature review and hypothesis development

### Human rights, business sectors, and sustainable development goals

Winkler and Williams [52] explained SDGs as a way to address the issues related to human dignity and equality. Although these are not outlined according to the human rights law, they work for humans, society, and the economy to achieve a more sustainable world. SDGs and their involvement with human rights will support them in framing the plans and programs in light of participation, equality, and accountability, promising no one leaving behind. Makarenko and Plastun [31] tried to clear the influence of SDGs in transforming the business process from conventional systems to modern sustainable practicing through incorporating the concepts of sustainability at all levels of the decision-making process and business activities. They mainly focused on sustainable reporting through which corporations identify key stakeholders, their rights, and information needs.

Being the main driver of productivity, business sectors can contribute to any SDG with their capabilities and resources. But there is a counter thinking that the UN agenda is convincing business organizations to join in the collaborative partnerships; simultaneously, it is making the corporations accountable for violating human rights that need to be crucially understood [16]. Violation of labor rights, failure to meet democracy, and enforcing human rights had lowered the level of achievements in SDGs in Indonesia during the Covid-19 pandemic. To avoid this kind of picture in future, strong enforcement of human rights and paying attention to detailed policy enforcement to reduce discrimination are needed during any type of pandemic and in normal situations as well. This will also create job security, improve economic growth and achieve SDGs [6]. Saha and Jannat [45] conducted a study on disclosures related to SDGs based on 16 recommendations based on four themes, including governance, strategy, management, and performance. They termed SDG as a roadmap for corporations that can help to make a long-term positive impact on stakeholders' minds and also on the environment. They found that most of the sampled companies are conscious of the SDGs, but they are unsure how to report, what, and how much to report. SDGs are actually working to ensure food availability, food sustainability for all, and global sustainability. The author showed an interrelationship between food access and waste, human rights, safe water and sanitation, SDGs, and social justice. Although SDGs are not clearly stating for the human right to food, they implore us to make a stronger stand for reducing food waste to avoid a manmade food crisis [15]. A relationship between human rights and foreign investment based on a case study between oil companies and the governments of Cameroon and Chad was presented by Sikka [41]. The author found limitations of conventional accounting for being not responsible for human rights and asked for counter accounts to challenge and reduce the domination of corporations and establish human rights. McPhail & Ferguson [32] identified key issues and themes of business and human rights and their relevance in accounting research. They tried to address the lack of engagement in the accounting profession and assurance service in the area of human rights.

### Disclosure on slavery and ownership structure

Schaper and Pollach [47] identified a set of measures that can be included in the reporting process to make the slavery reporting more substantive and transparent. It can be expected from their findings that there will be an urge for improved regulations and corporate disclosure practices. It is the firm that’s specific attributes worsen the organizational risk such as poor governance and internal control system [3]. Ownership structure is one of the major factors affecting voluntary disclosures as they affect the efficiency of the company and incentive system of managers [20]. Ownership structure can be comprises of institutional, foreign, family, sponsor and directors, controlling etc. [8]. Ismail et al. [24] investigated prior literature on corporate governance mechanisms, disclosure on human rights, and their impact on financial performance to find out the effect of non-financial disclosures on financial performance. They considered 11 human rights indicators, including training procedures, incidents of gender discrimination, child labor, forced labor, security practices, remediation policy, etc. Ikpor et al. [23] has found a negative relation between ownership structure and sustainability reporting suggesting that diversified ownership positively influence the voluntary reporting of the companies. On the other hand, Nguyen et al. [38], Onoja et al. [40] found a positive relationship between ownership structures and integrated financial reporting which is quite similar to sustainability reports. Maama,H. & Gani, S. [22] stated that institutional and individual ownership are more influential to non-financial reporting than firms with large scale of shareholders. Previous studies have established a link between ownership structure and sustainability reporting. However, we are interested in examining the relationship between slavery disclosure and ownership structure. Thus, we further hypothesize as:

- H1: Disclosure on Slavery (SDI) has a significant positive relationship with Directors Ownership (DOW).
- H2: Disclosure on Slavery (SDI) has a significant positive relationship with Institutional Ownership (IOW).
- H3: Disclosure on Slavery (SDI) has a significant positive relationship with foreign ownership (FOW).

### Disclosure on slavery and profitability

Crane et al. [13] outlined a framework of modern slavery that captures the important shapes of business models of contemporary slavery, explaining two forms: cost minimization-revenue generation and producer-intermediary. Their findings provided new insight into the protection of the business from the flaws of slavery, addressing viable threats coming from competitors exercising modern slavery and identifying risks that might be associated with modern slavery presented in the stages of supply chains. Micah et al. [33] tried to examine the impact of human resource disclosure on firm performance. They studied several variables related to human resource disclosure, such as a separate human resource statement, employment report, human resource policy, value creation, performance recognition, and fund for employees profitability measures, including return on asset and return on equity. They found a significant relationship between the disclosure index and performance measures indicating that each organization should take necessary measures to disclose its human resource information. Ali et al. [2] evaluated disclosure about human resources and analyzed the relationship between disclosure index and organizational attributes, including size, service length, profitability, number of employees, ownership structure and pages. They considered human resource statement, policy, training, and development, remuneration, retirement benefits, turnover, recruitment cost, performance recognition, etc., as indicators of human resource disclosure. They found an insignificant negative impact of profitability on human resource disclosures and recommended more emphasis on disclosing more information about human resources. On the other hand, Maama,H. & Gani, S [22], García-Sánchez et al. [18] and [26], found a significant positive relationship between sustainability reporting and profitability. However, our objective is to find out if there is any relationship between slavery disclosure and ROA. Thus, we further hypothesize as:

- H4: Disclosure on Slavery (SDI) has a significant positive relationship with Profitability (ROA).

### Disclosure on slavery and company age

In the era of a knowledge-based economy, each organization should disclose information about the human resource to stakeholders. It was found that larger companies are disclosing more information about human resources than smaller companies, financial companies are making better disclosure than non-financial companies, and a positive relationship between profitability and human resource-related disclosures. No relationship was found with age and disclosure index. Regulatory structures play a major role in determining the level of the disclosure; the strong the regulations, the better the disclosure is [1]. Dan Perbankan, J. K. [14] conducted a study on firm size, leverage, age, profitability and sustainable disclosure and found that firm age has a significant negative impact on sustainability reporting suggesting the latest firms are more interested in voluntary disclosures than older firms. On the other hand, Trencansky, D., & Tsaparlidis, D. [50] found a statistically insignificant impact of company age on sustainability issues. However, we are interested in examining the relationship between slavery disclosure and age. Thus, our study develops the following hypothesis:

- H5: Disclosure on Slavery (SDI) has a significant positive relationship with Company Age (AGE).

### Disclosure on slavery and leverage

Recent regulatory developments by United Nations are providing continuous guidelines for business and protecting human rights. Disclosing issues related to human rights has become a major part of corporate governance. Although corporations are now debating about human rights, they need to be more responsible by disclosing information related to social responsibilities, including human rights, to manage their relationship with stakeholders [33, 42, 47] Because of several incidents of corporate misbehaviors, there has been a decline in trustworthy relationships with the stakeholders, to gain and improve the trust again, the international organizations and policymakers suggested that corporations to work for ethics, sustainability, and responsibility. At the same time, they should maintain a communicative relationship with the stakeholders. Hardika et al. [21] stated that higher the leverage ratio the more the company will be intending to reduce costs that are incurred for voluntary disclosures such as sustainability reporting indicating a negative relationship between the two. In the same way, [14, 48] found a significant negative relationship between leverage and sustainability reporting. Previous studies have established a link between leverage and sustainability reporting. However, we are interested in examining the relationship between slavery disclosure and leverage. Thus, we posit the following hypothesis:

- H6: Disclosure on Slavery (SDI) has a significant positive relationship with Leverage (LEV).

### Disclosure on slavery and board size

Kusumastuti [27] analyzed the impact of company performance and company characteristics on human resource disclosure using secondary data and multiple regression analysis. They found that the banks with a longer life are disclosing less information than those with a smaller life span; this may be because they are experienced in financial reporting and think that it will not affect their profitability and investment. They also found that capital adequacy ratio, leverage, loan to deposit ratio, and board size have a positive and significant impact on human resource disclosure. [5, 6, 24, 29, 30, 49], found that board size, is significantly and positively correlated with sustainability reporting. On the other hand, Ganesan, Y. et al. [17] and Sarkar, S. H. [46] found insignificant relationship with board size and sustainability disclosure. In the study of Turkish enterprises, Önder, Ş., & Baimurzin, R [39]. found that sustainability disclosure decreases with the increase in board of directors. Previous studies have established a link between board size and sustainability reporting. However, we are interested in examining the relationship between slavery disclosure and board size. Thus, we further hypothesize as:

- H7: Disclosure on Slavery (SDI) has a significant positive relationship with Board size (BS).

## Research design and methodology

### Sample, and variables

There were 33 private commercial banks listed on Dhaka Stock Exchange, and we have selected 29 (87% of total study population) out of them (except four 4th generation banks) as study samples. We have selected only private-owned commercial banks of earlier generations because they have adopted modern technologies to provide better service earlier than others, and these banks carry a reputation for maintaining professionalism with their employees as well as customers. Annual reports from 2016 to 2021 were studied; although our target was to study up to 2022, due to the COVID pandemic, some of the annual reports of that year were not available. Data were collected during the beginning period of 2022. The study was done in light of SDG 8: Decent Work and Economic Growth, as it fully relates to our purpose of analyzing slavery reporting practice. Out of 10 targets, we have selected five namely 8.2, 8.5, 8.6, 8.7, and 8.8 based on disclosure uniqueness. Each annual report was studied thoroughly based on the following business reporting themes developed by **Global Reporting Initiatives** and **UN Global Compact, and a disclosure index** was prepared. Content analysis as it was one of the most used techniques in sustainable disclosures and some sub-themes were used to assess the nature, range, and quality of the reporting. Detailed disclosures on the following themes were examined based on annual reports of companies that were disclosing in more detail than others (Table 1).

**Table 1:** Assessment of dependent variable Disclosure on Slavery (SDI)

Targets	Themes	Weight	Disclosure examples
8.2	Training and education	1	Training type, scope, gender, employee category, and hours
8.5	Employment and workforce	1	The total number of employees, gender, employment type, etc.
	Remuneration and Benefit	1	Salary, health care facilities, insurance, provisions for retirement etc.
	Parental Leave	1	Number of employees (by gender) enjoying parental leave
	No Discrimination	1	A Policy of no discrimination regarding hiring, placement, promotion, termination, retirement, training etc.
	Working Hour	1	Working hour per week, including overtime
	Corporate Governance	1	Number of individuals by gender, age, minority, etc.
8.6	Employment Condition	1	The total number of new employees, employee turnover by age, and gender
8.7	Labor Right	1	Policy of labor right and standard
	Protection of children and young worker	1	Strong code for prevention and monitoring of child labor
	Abolition of child labor	1	Measures for effective abolition of child labor
	Elimination of forced labor	1	Measures to the effective elimination of forced labor
	Human Rights	1	Human rights compliance
8.8	Occupational Safety	1	Safety and health policies, compliance with safety policies
	Labor Relations	1	Available policy for labor rights awareness
	Labor practices in supply chain	1	Code and application of labor rights in supply chain

Formula of SDI: (Total score of individual company/ Maximum score) × 100

Total number of targets is 5, and themes are 16. Keywords that are used for investigating annual reports are training, the number of employees, remuneration, salary, benefits, parental leave, maternity, discrimination, equal rights, work hour, corporate governance, and employment conditions, labor rights, labor code/policy, forced labor, child labor, safety, health, supply chain, etc.

### Data analysis techniques regression model

Descriptive statistical tools were used to investigate the level of slavery disclosure, ownership, profitability, age, and leverage and board size over the period of year 2017–2021. To test the multicollinearity variance inflation factors (VIF) were computed. To determine the relationship between dependent variable and independent variables Pearson Correlation, Linear Regression and panel data analyses were conducted. The regression model is:

$$SDI_{{it}} = {\mkern 1mu} \alpha _{0} + \beta _{1} DOW_{{it}} + \beta _{2} IOW_{{it}} + \beta _{3} FOW_{{it}} + \beta _{1} ROA_{{it}} + \beta _{1} AGE_{{it}} + \beta _{1} LEV_{{it}} + \beta _{1} BS_{{it}} + \varepsilon _{{it}}$$

where, i = 1, …,29, t = 1 to 5, SDI- Disclosure on Slavery, DOW- Director’s Ownership, IOW- Institutional Ownership, FOW- Foreign Ownership, ROA- Return on Asset, AGE- Number of years of a company after the incorporation and BS-board size, respectively (Table 2).

**Table 2:** Assessment of independent variables

Director’s Ownership (DOW)	The proportion of the number of shares owned by directors/ sponsors to total outstanding shares
Institutional Ownership (IOW)	The proportion of the number of shares owned by institution to total outstanding shares
Foreign Ownership (FOW)	The proportion of the number of shares owned by foreign owner to total outstanding shares
ROA	Earnings after Tax & Interest divided by Total Assets
Age	Number of years of a company after the incorporation
Leverage (LEV)	Total Debt divided by Total Equity
Board Size (BS)	Number of members in the board

## Results and discussions

From the content analysis, we have found the nature and extent of slavery reporting. All of the companies were aware of modern slavery as there was no company with no disclosure. Minimum three components were found to be disclosed by only one company in 2017. But this company showed the highest number of disclosed components in 2021, i.e., 15 out of 16. This indicates that 2017 was the beginning period of reporting SDGs, and the concern to report was increasing day by day. The full disclosure was done by Mercantile Bank in 2021, and the average disclosure was in the year 2018, indicating it was a year of rising consciousness about slavery reporting or SDGs reporting (Tables 3, 4).

**Table 3:** Descriptive statistics of total disclosure by each company in each year

Variable	Obs.	Mean.	Std. Dev.	Min.	Max.
Year	174	2018	1.712754	2016	2021
Total Disclosure	174	0.6332	0.19409	3	16

Total market capitalization of 29 companies was Tk.6630.295 million

In Table 4, 16 components under disclosure of slavery (SDI) with basic statistics in five years individually was presented. There visualized a clear scenario that average of these 16 components increases slightly as time increases that signifies that our listed commercial banks were performing well and trying to accomplish SDG goal under the monitoring of good governance. On the contrary, out of sixteen components, only three (training and education, employment and workforce and remuneration and benefit) anchored 100% on each year. On Average, within 80 to 99%, five components (Corporate governance, Human Rights, Occupational safety, no discrimination and Employment condition) were fulfilled. So, in a nutshell, these eight pillars were prioritized more than the rest of the half. On the other hand, these eight component’s average were greater than grand average (63%). The poorest performance was identified on parental leave as its average observed only 11%. The others three weaker component were working hour, elimination of forced labor and labor practices in supply chain as their average ranges below 30%. There was an unavoidable fact that though banks have a rule for 8 h of working per day and 5 days per week, very few banks were disclosing information about this. It had been also a regular picture in Bangladesh that bankers were working more than the maximum working hour, which was more regular in branches situated in the urban area. The average number of companies disclose information about labor rights and relations, and they have a policy or code to protect labor rights in Bangladesh. The Bangladesh Labor Code 2006 was followed by all companies. The number of companies disclosing information about labor practices in the supply chain was low during the first two years of the study, and the number was increasing in the latest years. The others tool, such as standard deviation (SD), minimum (Min.) and maximum (Max.) was provided.

**Table 4:** Disclosure of SDI by each component along with basic statistics

Components of SDI	2016	2017	2018	2019	2020	2021	Average in percentage
Training and education	29	29	29	29	29	29	100%
Employment and workforce	29	29	29	29	29	29	100%
Remuneration and Benefit	29	29	29	29	29	29	100%
Corporate Governance	28	29	29	29	29	29	99%
Human Rights	27	27	29	29	29	29	98%
Occupational Safety	23	25	28	29	29	29	94%
No Discrimination	24	24	25	26	26	26	87%
Employment Condition	22	22	25	26	26	26	84%
Labor Right	11	11	15	17	18	18	52%
Labor Relations	11	12	14	18	18	18	52%
Protection of children and young worker	7	7	9	14	14	14	37%
Abolition of child labor	6	6	9	14	15	15	37%
Working Hour	6	6	8	9	10	10	28%
Elimination of forced labor	5	5	7	9	10	10	26%
Labor practices in supply chain	2	2	5	10	10	10	20%
Parental Leave	2	2	2	4	6	6	12%
	*Basic statistics*						
Average	16.31	16.56	18.25	20.06	20.44	20.44	
SD	10.84	10.98	10.44	9.10	8.64	8.64	
Min.	2	2	2	4	6	6	
Max.	29	29	29	29	29	29	

Table 5 provides an overview about the independent variables of the study. As this study executes 29 commercial private-owned banks and study period is observed for six years (2016–2021), hence, sample size comes to 175. As index is ranges from 0 to 1, the average of outcome variable (index) was 0.632, which was slightly high than other three ownerships. The average of ROA clogs with zero resulting for profitability within five years of study sample was not satisfactory. The average of three crucial control variables was quite larger. In brief, average age of each institution was 27 years, whereas leverage (LEV) and board size (BS) were 13.21 and 13.84, respectively. In case of measures of dispersion, standard deviation was analyzed. Four indices (including outcome variable) were closes to each other but not for control variables. In case of maxima and minima, there was some interesting scenario for two articular variables- return on asset (ROA) and leverage (LEV). Hence, profitability of particular bank faces losses and buying security forces in risk. As both variables face negativity, the maximum leverage value or average value of the leverage also indicates highly leveraged industry. The maximum size of the board was 22 and minimum was 6.

**Table 5:** Descriptive statistics of all variables

Variables	Obs	Mean	Std. Dev.	Min.	Max.
Index	174	0.63	0.19	0.19	1.00
DOW	174	0.39	0.14	0.04	0.87
IOW	174	0.20	0.095	0.03	0.5156
FOW	174	0.34	0.07	0.00	0.44
ROA	174	0.01	0.049	− 0.042	0..640
AGE	174	27.22	7.83	16.00	46.00
LEV	174	13.21	4.66	− 2.22	27.23
BS	174	13.84	4.20	6.00	22.00

Table 6 represents the Pearson correlation matrix for dependent and independent variables. All variables have a positive relationship with dependent variable except return on asset (ROA), age (AGE) and leverage (LEV). Both of ROA and LEV showed insignificant relationship. A negative correlation between age and index indicates that young companies have a greater score than the old companies, which means new companies were more concerned about SDGs than older companies. Moreover, the negative correlation between LEV and index indicates that highly leveraged companies were disclosing less than the others. Out of three ownership indices, DOW and IOW performed weekly as their correlation coefficient were neither strong nor statistically significant. In contrast, foreign ownership (FOW) correlated at 5% level of significance as the correlation coefficient came 0.316. On the other side, return on asset (ROA) correlates positively with Leverage (LEV) at 5% level of significance and leverage (LEV) negatively correlated with age (AGE) at 5% level of significance. Overall, Foreign Ownership (FOW) is positively correlated with SDI as external capital creates pressure to maintain sustainable issues; board size (BS) is positively correlated with SDI proving that higher number of board members can supervise better from different angles with their experience and learning. The test of multicollinearity shows that all of the independent variables are free from multicollinearity.

**Table 6:** Correlation matrix and test of Multicollinearity

Variables	SDI	DOW	IOW	FOW	ROA	AGE	LEV	BS	VIF
SDI	1								
DOW	0.130	1							1.38
IOW	0.028	− 0.435**	1						1.37
FOW	0.316**	0.016	− 0.255**	1					1.18
ROA	− 0.085	− 0.18	− 0.046	0.003	1				1.15
AGE	− 0.275**	− 0.250**	0.062	− 0.030	− 0.055	1			1.10
LEV	− 0.044	− 0.063	0.052	− 0.058	0.260**	− 0.221**	1		1.10
BS	0.285**	− 0.075	− 0.042	− 0.075	0.018	− 0.145	0.222**	1	1.09

(*** and ** represents 1% and 5% level of significance, respectively)

From the following tables (Tables 7 and 8), it can be seen that there was mysterious output of each method as compared to other. Four methods- OLS (Ordinary Linear Square), pooled ordinary square (POLS), fixed effect (FE) and random effect (RE), respectively. Under OLS or multiple linear regression model (MLR), all variables effects with statistical evidence. However, to find out the within effects and omitted biasness panel data estimations FE and RE were adopted. Out of three ownership indices, all significantly impacted positively; institutional ownership and foreign ownership (FOW) impacted strongly (*β* = 0.518 and *β* = 0.967, respectively). This is because external capital flow create a substantial pressure on companies to behave in a more socially responsible way. The relationship between ROA and SDI showed an insignificant result. Leverage was meagerly influenced by SDI and AGE has a strong negative impact on SDI suggesting that new companies are more concerned about slavery issues, age is not a matter for it. On the other hand, for pooled ordinary least square (POLS), four indices and four control variables impacted with statistical evidence. Specifically, as one-unit increase in institutional ownership (IOW), SDI will increase by 0.517 with 1% level of significance. Like as before, ROA insignificantly associated with SDI in negative sign. Now for panel data analysis, fixed effect (FE) and random effect (RE) models were examined. In FE, only Leverage (LEV) of firm effected negatively with SDI. But on the other hand, random model visualized positive effect on SDI. In case of ownership indices, institutional ownership (IOW) and foreign ownership (FOW) effect positively on SDI with 1% level of significance. Board Size also depicts a significant positive relationship with SDI at 5% level of significance. In case of diagnostic test, R-square, Adjusted R-square and F-statistic examined out of these four models, fixed effect model exhibited better. For FE and RE, between sum of square (BSS) and within sum of square (WSS) was shown (Table 9).

**Table 7:** Linear, POLS, and cross section fixed effect tests results

	OLS	POLS	FE	RE
DOW	0.268*	0.268**	0.258	0.316**
IOW	0.518***	0.517***	0.307	0.460***
FOW	0.967***	0.966***	0.839***	0.930***
ROA	− .174	− 0.174	0.013	0.048
AGE	− .006***	− .005***	0.013**	0.0005
LEV	− .005	0.016	− 0.001	0.0004
BS	.016***	0.016***	0.005	0.114**
Constant	0.394***	0.393***	0.034	0.203
R^2^	0.575	0.33	0.790	0.115
Adj. R^2^	0.302	0.30	0.736	0.07
F-statistic	11.493***	11.49***	14.529***	3.03**
DW-statistic	0.824	0.503	1.42	1.187
SS(between)	–	–	0.0003	0.3172
SS(within)	–	–	0.114	0.0647

(*, **and *** represents 10%, 5% and 1% level of significance respectively)

Statistical tools: Eviews 10

**Table 8:** Likelihood ratio and hausman test

Test summary	Statistic	d.f	Prob.
Cross section F	10.567	− 28.135	0.0000
Cross section Chi-square	198.453	28	0.0000
Cross section Random	15.108	7	0.063

**Table 9:** Decisions of Hypothesis Testing

H1	DOW → SDI	Supported
H2	IOW → SDI	Supported
H3	FOW → SDI	Supported
H4	ROA → SDI	Not supported
H5	AGE → SDI	Not supported
H6	LEV → SDI	Not supported
H7	BS → SDI	Supported

From the likelihood ratio test, we see that probability is zero rejecting the null hypothesis, which says the fixed effect model is more appropriate than POLS in this dataset. And from the Hausman test, the probability is greater than 0.05 indicating the random effect model was appropriate for this data set. Between linear regression and the cross section fixed effect model, the later gave the most significant results, but the former provided a higher R square value than the later.

Further, the current study linked ownership structure with slaver disclosure and findings are in line with [22, 38, 39] showing ownership structure exhibits significant positive relationship with slavery disclosure. These findings suggest that corporations in Bangladesh can welcome high foreign and institutional shareholdings to meet their accountabilities to stakeholders and attain sustainable goals. However the result is not consistent with some earlier results [23] related with sustainability reporting. In case of profitability and slavery disclosure index, our results found insignificant relationship with slavery disclosure which is consistent with [2] and doesn’t match with the findings of [18, 22, 26]. However, more research is needed to find a strong direction between this relationships. The result of age and slavery disclosure supported [1, 50], and in against with [14]. The explanation behind this can be that the length of service is not matter for the companies to disclose slavery information, more regulations and consciousness regarding sustainable working environment are the main influencing factors. The relationship between leverage and slavery disclosure are not consistent with previous studies [14, 21, 48] suggesting that more research is needed to depict a clear picture between this two. Finally, we found that board size has a positive impact on slavery disclosure which is consistent with [5, 6, 24, 29, 30, 49] suggesting that an increased number of board members enhance the consciousness about slavery and sustainability.

## Conclusion

### Summary and policy implications

There are several cases of the corporate environment, misbehaviors, and safety that reduce the credibility of the relationship between corporations and stakeholders. To maintain a healthy relationship with all stakeholders, companies must work as strong players in sustainable development. The need for engaging SDGs, especially human rights, environment, labor protection, and anti-corruption, into strategies and governance of companies has been addressed by national and international policymakers, associations, and government bodies. This will benefit the stakeholders in evaluating their interested companies, whether the companies are merely disclosing information or they are serious about the protection of the rights of stakeholders. In this way, the process will add value to the profitability and reputation of the company. The aftershock of the pandemic will majorly impact the global economy, community development, education, etc. There is also a rising concern for protecting human rights in times of crisis [9].

This study has strong policy consequences on banking sector as well as all other reporting companies in Bangladesh. The organizations should work to increase consciousness about slavery accounting by recognizing the benefit of reporting non-financial information, such as information about human resources, environmental factors, social factors, etc. As this type of disclosure is voluntary, it is necessary to shift it to a mandatory disclosure requirement. Previous studies in related fields provided evidence of positive and negative correlation among the selected variables; the regression analysis of this study also depicted the same results. Although it is tough to determine the immediate effect of this type of reporting on performance, a small change of improvement can be seen, it is a matter of time to build a relationship of reliability and credibility with the stakeholders, especially with investors and customers.

### Limitations and further research

This study is limited by the sample of analysis and timeframe, as some companies are not disclosing their latest annual reports on websites. There are strict guidelines for reporting for the banking industry, but the picture may not be the same for other industries. Further research should be run, including more companies from different industries from different countries to compare disclosure on slavery accounting. This research is exploratory; it will work as a guide for further research on slavery accounting with an increased number of indicators and variables.

## Data Availability

The data will be provided on request.

## Abbreviations

SDGs: Sustainable development goals
MDGs: Millennium development goals
ROA: Return on assets
SDI: Disclosure on slavery
LEV: Leverage
FOW: Foreign ownership
DOW: Directors’ ownership
IOW: Institutional ownership
BS: Board size
OLS: Ordinary linear square
POLS: Pooled ordinary square
FE: Fixed effect
RE: Random effect
vif: Variance inflation factor
